# Fatigue Tests and Analysis on Welded Joints of Weathering Steel

**DOI:** 10.3390/ma15196974

**Published:** 2022-10-08

**Authors:** Rongrong Sheng, Yuqing Liu, Ying Yang, Rui Hao, Airong Chen

**Affiliations:** 1 Department of Bridge Engineering, Tongji University, Shanghai 200092, China; 2 Anshan Iron and Steel Group Co., Ltd., Anshan 114021, China; 3 Department of Civil Engineering, Aalto University, 02150 Espoo, Finland

**Keywords:** weathering steel, welded joints, fatigue performance, fatigue tests, numerical analysis

## Abstract

To investigate the fatigue performance of vertical web stiffener to deck plate welded joints in weathering steel box girders, six specimens of the weathering steel (WS) Q345qNH, four specimens of WS Q420qNH, and four specimens of the plain carbon steel (CS) Q345q for comparison were tested by a vibratory fatigue testing machine, considering different steel grades, yield strengths, stiffener plate thicknesses, and weld types. The fatigue strength was evaluated based on *S*-*N* curves and the crack propagation was analyzed by linear elastic fracture mechanics (LEFM). The results show that the fatigue crack of the welded joints was initiated from the end weld toe of the deck plate and subsequently propagated both along the thickness of the deck plate and in the direction perpendicular to the stiffener plate. The fatigue crack initiation and propagation life of WS Q345qNH specimens were longer than those of CS Q345q specimens. The fatigue crack propagation life of WS Q345qNH specimens was longer than that of WS Q420qNH specimens, while the initiation life bore little relationship to the yield strength. Increasing the stiffener plate thickness effectively delayed crack initiation and slowed down its propagation. Compared with fillet welds, full penetration welds extended the fatigue crack propagation life, while no significant improvement was implied for the initiation life. The WS and CS specimens could be classified as having the same fatigue strengths by nominal stress, hot spot stress, and effective notch stress approaches, which were *FAT* 50, *FAT* 100, and *FAT* 225, respectively. Meanwhile, their material constants for LEFM were relatively close to each other.

## 1. Introduction

**Figure 1:**
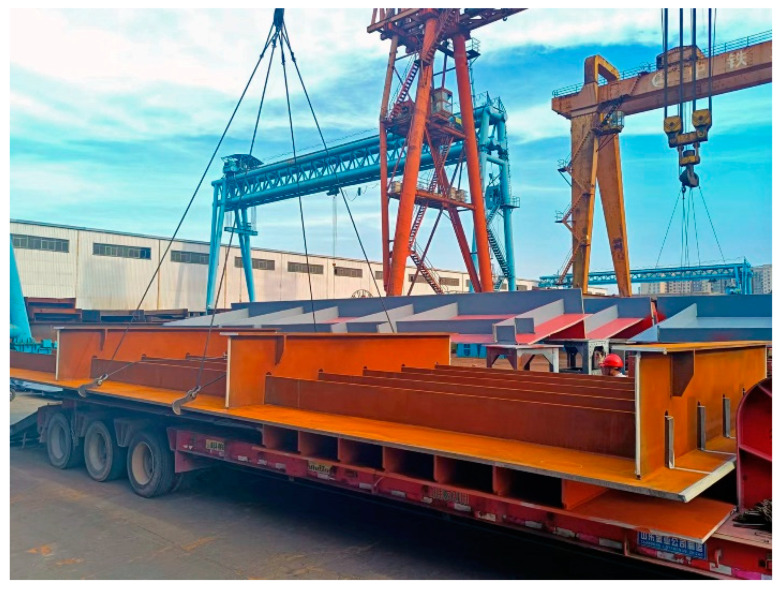
Orthotropic steel deck of weathering steel.

Weathering steels (WS) are low-alloy steels with the addition of alloying elements, such as Cu, Cr, Ni, P, Si, and Mn. The introduction of those alloying elements can facilitate the formation of a dense and strongly adherent rust layer during wet/dry cycles. Compared to plain carbon steels (CS), the corrosion resistance of WS is enhanced due to the protective rust layer [1,2,3,4]. This contributes to the application for WS in bridge structures (e.g., orthotropic steel decks) (Figure 1).

The fatigue performance of WS has been investigated by many researchers. In the aspect of material fatigue of WS, Chen et al. [5] obtained the fatigue limit and the crack growth rates of ASTM A709 HPS 485W steel through testing on flat sheet specimens and single-edged tension specimens. Su et al. [6] investigated the fatigue crack growth thresholds and fatigue crack growth rate parameters of Q345qDNH steel by compact tension specimens. For constructional details of WS, Albrecht et al. [7,8] carried out fatigue tests of a transverse stiffener detail to determine the effect of weathering time and exposure conditions on the fatigue life. Yamada and Kikuchi [9] examined the fatigue behavior of weathered transverse stiffener specimens and longitudinal gusset specimens. Su et al. [10,11] conducted fatigue tests of uncorroded butt joints and fillet welded joints to obtain the *S*-*N* curves, and discussed initial crack parameters for the numerical simulation of fatigue crack propagation. For structural components of WS, Albrecht et al. [12,13] explored the effect of exposure conditions and testing environments on the fatigue behavior of rolled or welded I-beams. Sause et al. [14] provided the design *S*-*N* curve and fatigue limit of uncorroded corrugated web I-girders. Vertical web stiffener to deck plate welded joints, as a typical detail in steel box girder bridges, are vulnerable to fatigue cracking [15,16]. When wheel loads act on them, bending stress is generated in the deck plate and stress concentration is caused at the end weld. This stress state is different from that of welded joints with longitudinal gussets, and therefore needs to be examined. Moreover, the relationship between structural parameters and the fatigue performance remains to be revealed.

The methods of fatigue analysis mainly include *S*-*N* curve methods and fracture mechanics methods. In *S*-*N* curve methods, structural details are classified into several or single fatigue strength categories represented by certain *S*-*N* curves. According to the reference stress, *S*-*N* curve methods can be divided into the nominal stress approach, the hot spot stress approach, and the notch stress approach. The nominal stress approach has the advantage of being easy to understand and operate for fatigue design practice, for which it is widely adopted in existing standards and specifications of steel structures [17,18,19]. However, its scope is within the categorized structural details, namely that it may not be applicable to novel or complex details. In the hot spot stress approach [20] and the notch stress approach [21], there are more general detail categories and fewer *S*-*N* curves than in the nominal stress approach. Both the approaches are well applicable to fatigue assessment of welded joints. But their calculation seems more complicated. Moreover, the hot spot stress is not defined at weld roots and the notch stress cannot be directly measured. Fracture mechanics methods [22] are able to establish the relationship between fatigue life and crack sizes, evaluate the influence of initial defects and the residual life of cracked components, but they are demanding in theoretical knowledge and calculating work. Besides, the initial crack size and material constants for computation need to be verified by experimental data. Research on the fatigue performance of WS structural details has mainly focused on the nominal stress approach.

In this paper, fatigue tests were conducted to investigate the fatigue performance of vertical web stiffener to deck plate welded joints of WS. Six specimens of WS Q345qNH, four specimens of WS Q420qNH, and four specimens of CS Q345q for comparison were tested by a vibratory fatigue testing machine, considering different steel grades, yield strengths, stiffener plate thicknesses, and weld types. Cyclic bending stress in the deck plate was applied to simulate the action of wheel loads. After that, the fatigue strength was evaluated with the nominal stress approach, the hot spot stress approach, and the notch stress approach, respectively. The fatigue crack propagation was analyzed by linear elastic fracture mechanics (LEFM) as well. Stress intensity factors were computed by the extended finite element method (XFEM). The fatigue crack propagation rates were obtained. And the material constants of fatigue crack propagation were estimated.

## 2. Materials, Methods and Experiments

### 2.1. Specimens

As shown in Table 1, 14 test specimens of vertical web stiffener to deck plate welded joints were designed and fabricated. Among them, W stood for WS specimens and C for CS specimens. To examine the relationship between structural parameters and fatigue performance, the varied parameters included steel grades, yield strengths, stiffener plate thicknesses, and weld types. The chemical compositions of the steel grades are listed in Table 2.

**Figure 2:**
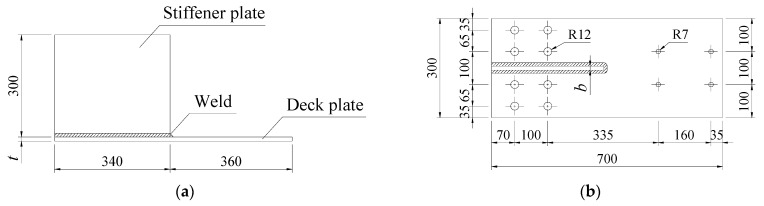
Configuration of test specimens (mm). (**a**) Front view. (**b**) Top view.

Figure 2 shows the configuration of the test specimens, where the holes with a radius of 12 mm were used to fix the specimens to a pedestal and those with a radius of 7 mm were for installing a vibration motor. The specimens were processed by CO_2_ welding. After welding, magnetic particle flaw detection and ultrasonic flaw detection were carried out to ensure the welding quality.

### 2.2. Test Setup

**Figure 3:**
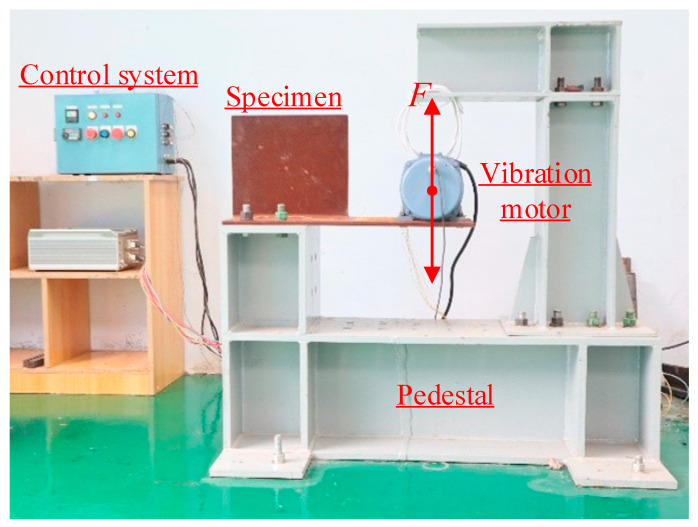
Test setup.

As shown in Figure 3, the specimens were tested by a vibratory fatigue testing machine [24]. They were fixed to the pedestal by bolts. The vibration motor was installed on the deck plate, which could generate a centrifugal force and cause bending stress in the deck plate to simulate the action of wheel loads. The applied stress range was adjustable through changing the rotor shaft rotation speed. Constant amplitude fatigue loading was adopted for all the specimens. The rotor shaft rotation speed was set to make the measured nominal stress range reach 80 MPa before the tests started, and afterwards remained unchanged, which meant that the stress ratio was kept at −1 during the tests.

### 2.3. Instrumentation

**Figure 4:**
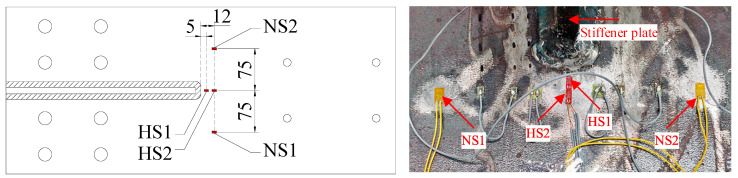
Arrangement of strain gauges (mm).

Strain gauges were used to monitor the strain history throughout fatigue crack initiation and propagation. Their arrangement is illustrated in Figure 4. Strain gauges NS1 and NS2 were set for measuring the nominal strain. The stress where they were attached was found to be equal to that at the end weld of the joints without a vertical stiffener, which excluded geometric stress concentration due to the vertical stiffener [24]. Strain gauges HS1 and HS2 were for obtaining the hot spot stress. They were located 5 mm and 12 mm from the end weld toe, in accordance with the recommendations of the type a hot spot linear extrapolation by the International Institute of Welding (IIW) [20]. The nominal stress range $\Delta \sigma_{\mathrm{nom}}$ and the hot spot stress range $\Delta \sigma_{\mathrm{hs}}$ are calculated by:

(1) $$\Delta \sigma_{\mathrm{nom}}=E\cdot (\Delta \varepsilon_{\mathrm{NS}1}+\Delta \varepsilon_{\mathrm{NS}2})/2,$$

(2) $$\Delta \sigma_{\mathrm{hs}}=E\cdot (1.67\Delta \varepsilon_{\mathrm{HS}1}-0.67\Delta \varepsilon_{\mathrm{HS}2})$$

where $\Delta \varepsilon_{\mathrm{NS}1}$, $\Delta \varepsilon_{\mathrm{NS}2}$, $\Delta \varepsilon_{\mathrm{HS}1}$, and $\Delta \varepsilon_{\mathrm{HS}2}$ are the strain ranges at NS1, NS2, HS1, and HS2, respectively; *E* is the Young’s modulus of steels and equal to 206 GPa.

### 2.4. Fatigue Crack Measurement

#### 2.4.1. Surface Crack Monitoring

**Figure 5:**
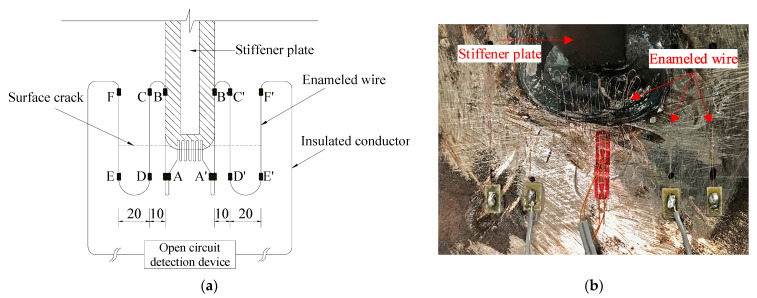
Surface crack monitoring. (**a**) Control circuit (mm). (**b**) Arrangement of enameled wires at the end weld.

As shown in Figure 5, a control circuit was used to monitor the surface crack initiation and propagation. It was composed of enameled wires with a diameter of 0.04 mm, insulated conductors and an open circuit detection device. A (A’), B (B’), C (C’), D (D’), E (E’), and F (F’) denote terminals. The enameled wires connected A to A’, A to B (A’ to B’), C to D (C’ to D’), and E to F (E’ to F’) and were glued onto the deck plate crossing the potential fatigue crack. A closed circuit was formed by the enameled wires, insulated conductors and the open circuit detection device in series. Once the crack propagated to the position of any enameled wire, the wire would be broken, and the circuit disconnected. Meanwhile, the open circuit detection device output a signal to the fatigue testing control system to stop the vibration motor. The crack length and the number of cycles at this time were recorded. After that, the broken wire was replaced with an insulated conductor. The open circuit detection device got reset, and fatigue loading resumed.

When the crack was initiated at the end weld and broke the enameled wire connecting A to A’, the number of cycles was recorded as $N_{\mathrm{toe}}$. Similarly, when the crack propagated to the edges of the weld and subsequently 10 mm, 30 mm away to break the wires connecting A to B (A’ to B’), C to D (C’ to D’), and E to F (E’ to F’), the numbers of cycles were taken as $N_{b}$, $N_{10}$, and $N_{30}$, respectively. After the E to F (E’ to F’) wire got disconnected, fatigue testing was terminated.

#### 2.4.2. Beach Mark Testing

**Figure 6:**
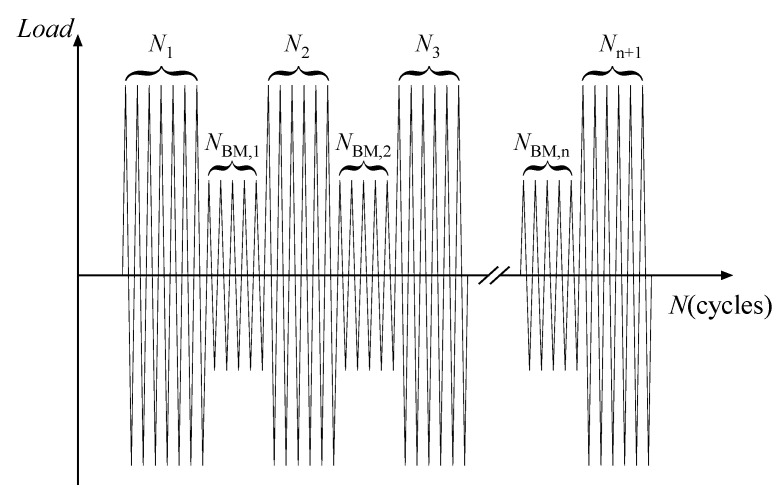
Schematic load sequence.

To track the propagating process of fatigue cracks along the length and depth, beach mark testing was carried out on some of the specimens, W1-2, W3-2, W4-2, W5-2, C1-1, and C2-2. During fatigue loading, fatigue crack propagation rates varied with the applied stress ranges, and the characteristics of the fracture surface changed accordingly. Therefore, alternate light and dark bands on the fracture surface, namely beach marks, could be generated by a certain load sequence to demonstrate the crack propagation. A schematic load sequence was given in Figure 6. In the fatigue tests of this research, the load ranges were reduced by half every $N_{i}$ cycles and lasted for about 100,000–140,000 cycles. The total number of cycles of beach mark testing $\sum N_{\mathrm{BM},\;i}$ was not included in the fatigue life. The specimens were eventually cut to expose the fracture surface, and the beach marks were observed and measured.

## 3. Test Results

### 3.1. Failure Mode

**Figure 7:**
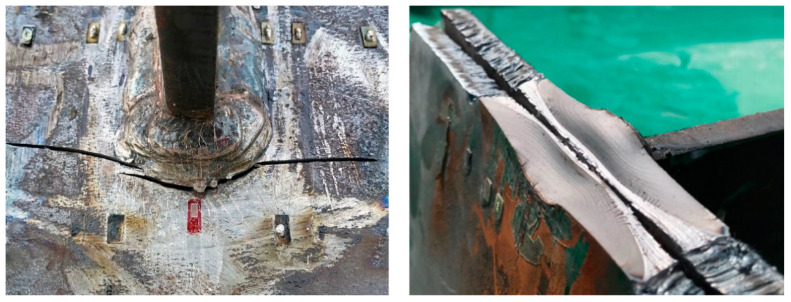
Failure mode.

Figure 7 shows the failure mode of the specimens. The fatigue crack of the welded joint was initiated from the end weld toe of the deck plate, and subsequently propagated both along the thickness of the deck plate and in the direction perpendicular to the stiffener plate. Finally, it almost penetrated through the deck plate. Besides, the crack shape appeared semi-elliptical on the fracture surface.

### 3.2. Stress Range

**Figure 8:**
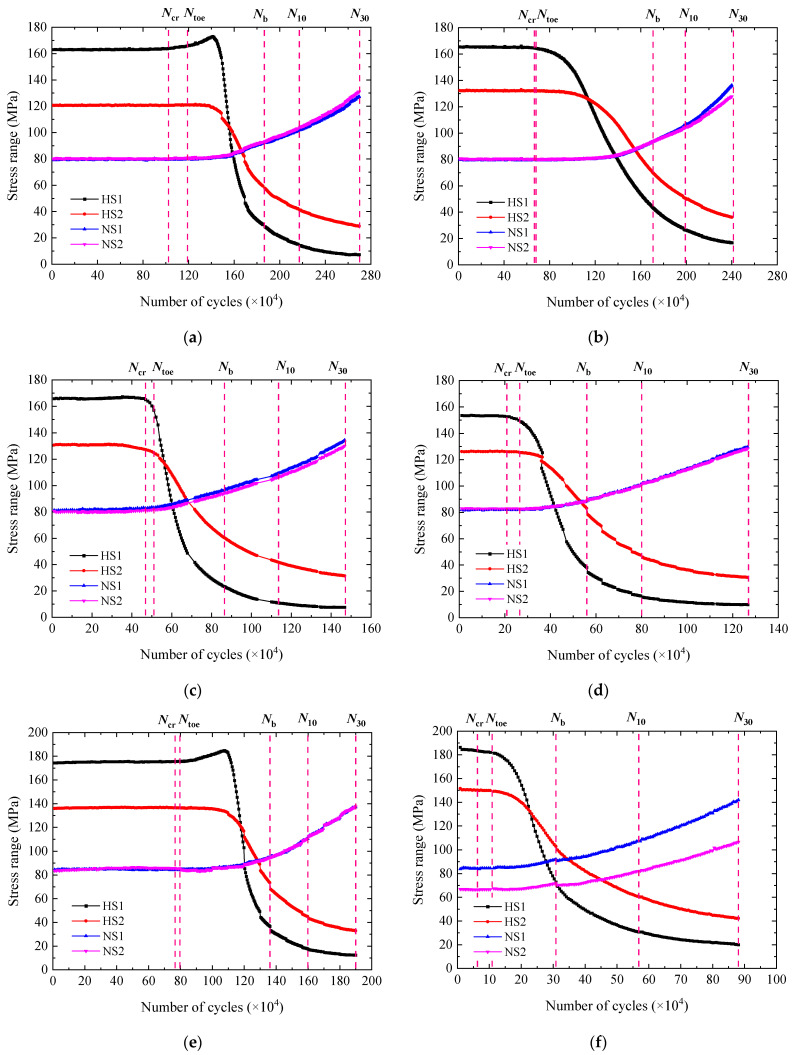

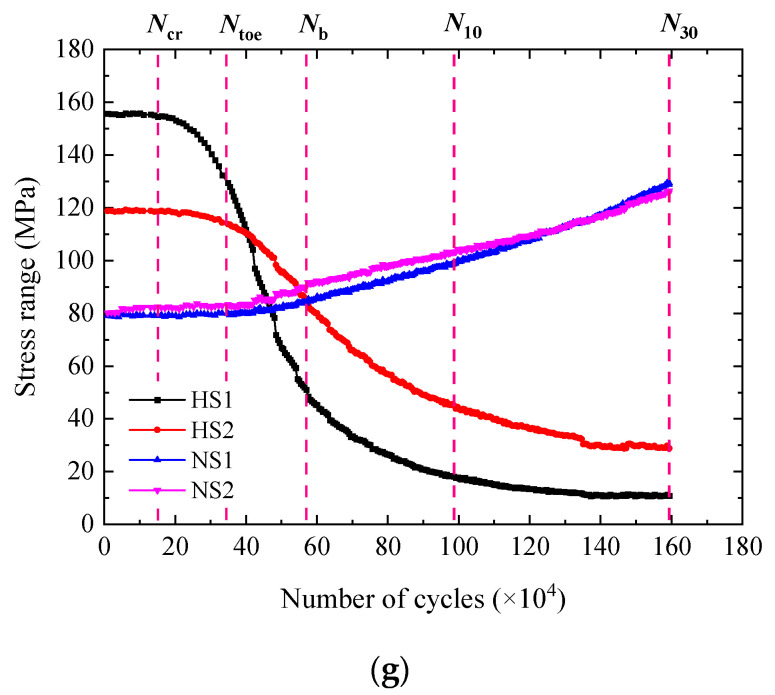
Variation of stress range with number of cycles for WS specimens. (**a**) W1-2. (**b**) W2-1. (**c**) W3-1. (**d**) W3-2. (**e**) W4-2. (**f**) W5-1. (**g**) W5-2.

**Figure 9:**
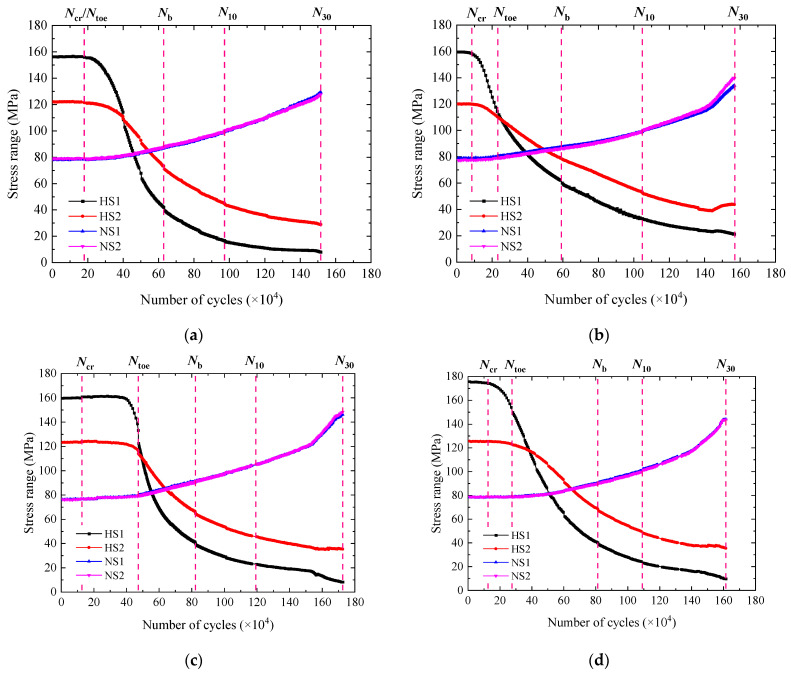
Variation of stress range with number of cycles for CS specimens. (**a**) C1-1. (**b**) C1-2. (**c**) C2-1. (**d**) C2-2.

**Figure 10:**
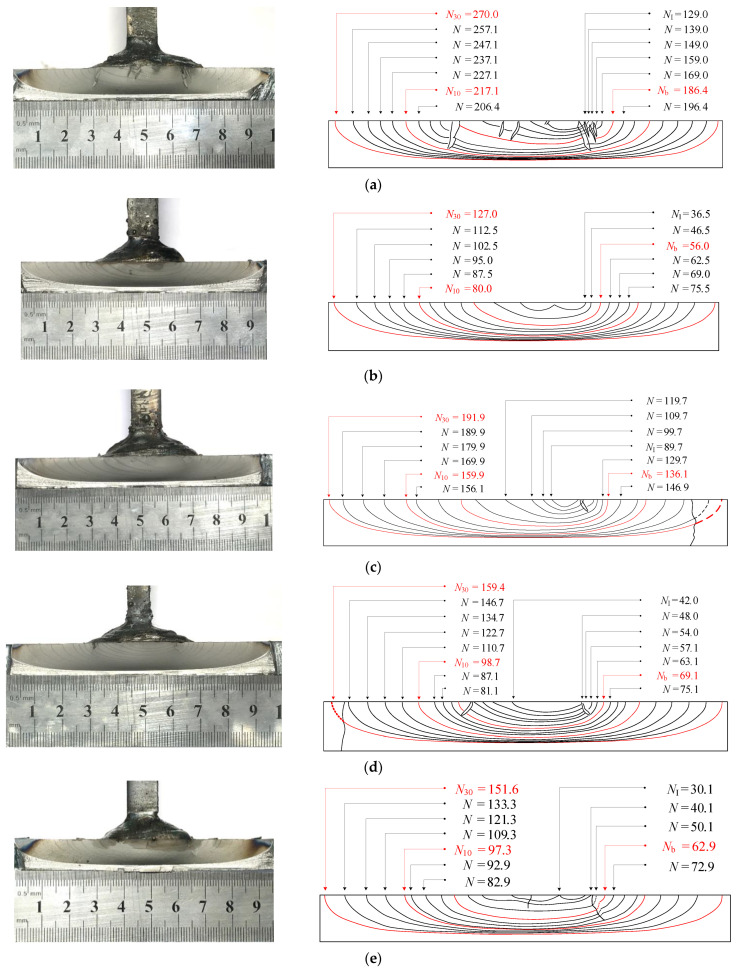

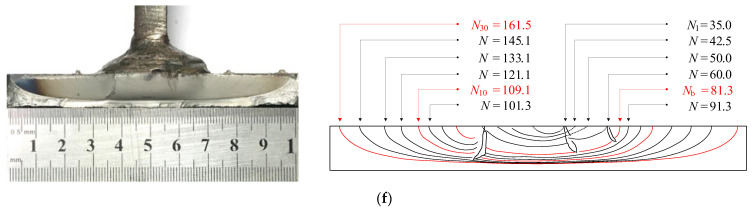
Beach marks on fracture surfaces (*N*: $\times 10^{4}$ cycles). (**a**) W1-2. (**b**) W3-2. (**c**) W4-2. (**d**) W5-2. (**e**) C1-1. (**f**) C2-2.

Figure 8 and Figure 9 show the variation of the stress range throughout the fatigue tests. For all the specimens except W1-2, W4-2, and C2-1, the stress range at HS1 and HS2 was first stable and went down thereafter. The decrease in the stress range at HS1 and HS2 resulted from the stress release in the vicinity of HS1 and HS2 after the fatigue crack was initiated from the end weld. The stress range at NS1 and NS2 showed a process of being stable first and rising afterwards. It was because the stiffness of the deck plate cross section was reduced due to the cracking and that the load on both sides increased consequently. For W1-2, W4-2, and C2-1, the stress range at HS1 rose slightly before its decrease. It was found that the fatigue crack was initiated from the side of the end weld toe but not the center, see the beach marks in Figure 10. The local stiffness was reduced, and the load distributed in the adjacent region increased. Therefore, the stress range at HS1 went up to some degree. It was reasonable to infer that the crack initiation of C2-1 was similar to that of W1-2 and W4-2.

It can be seen that the stress range at HS1 changed most with the number of cycles among the data at different gauges, which indicated that it was highly sensitive to cracking. The first inflection point of the stress range versus number of cycles curve of HS1 was designated as $N_{\mathrm{cr}}$, representing the fatigue crack initiation life. The wire connecting A to A’ at the end weld was spaced about 1.5 mm apart. It wouldn’t be broken until the fatigue crack grew long enough. $N_{\mathrm{toe}}$ was generally later than $N_{\mathrm{cr}}$. For that reason, $N_{\mathrm{cr}}$ was taken for the determination of the fatigue crack initiation life.

According to the stress range versus number of cycles curve of HS1, the fatigue testing could be divided into three stages. They were as follows. Stage I was the period from the start to $N_{\mathrm{cr}}$, where the crack was gradually initiated but the stress range remained stable. Stage II was from $N_{\mathrm{cr}}$ to $N_{b}$, where the crack grew rapidly and caused the stress range to fall sharply. Stage III was from $N_{b}$ to $N_{30}$. In this stage, the crack growth became slow and the stress range decreasing also slowed down. The total number of cycles of stage I and II was designated as $N_{\mathrm{CP}}$, representing the fatigue crack propagation life. $N_{30}$ denotes the total test life. It should be noticed that the stress range at HS1 mainly implied the fatigue cracking along the depth.

### 3.3. Fatigue Life

The fatigue test results are listed in Table 3. If the measured strains were unchanged and the enameled wires were not broken after the number of cycles reached 2 million, it was considered to run out. The run-out specimens were not included in the fatigue life analysis. Although $\Delta \sigma_{\mathrm{nom}}$ for W5-1 was 5.4% smaller than the expected value of 80 MPa, the deviation for the other specimens was within 5.0%. The stress concentration factor $K_{s}$ was the ratio of $\Delta \sigma_{\mathrm{hs}}$ to $\Delta \sigma_{\mathrm{nom}}$, which reflected the stress raising effect of the geometric discontinuity of vertical web stiffener to deck plate welded joints. It ranged from 2.08 to 2.74 and had little correlation with weld types and stiffener plate thicknesses. The ratio of $N_{\mathrm{CP}}$ to $N_{30}$ was designated as $R_{\mathrm{CP}}$, indicating how much the fatigue crack propagation life accounted for of the total test life. When stiffener plate thicknesses were the same, $R_{\mathrm{CP}}$ of WS specimens were less than CS ones.

As shown in Table 4, the specimens were compared with each other to analyze the effect of each structural parameter on the fatigue life. The structural parameters included steel grades, yield strengths, stiffener plate thicknesses and weld types. If the data of any specimen in group A were greater than those in group B, it was recorded as A > B. If the data of any specimen in group A were less than those in group B, it was recorded as A < B. Otherwise, it was recorded as A~B. The comparisons suggested that both steel grades and stiffener plate thicknesses had significant effect on the fatigue life of the welded joints. The fatigue crack initiation and propagation life of WS Q345qNH specimens were longer than those of CS Q345q specimens. The fatigue crack propagation life of WS Q345qNH specimens was longer than that of WS Q420qNH specimens, while the initiation life bore little relationship to the yield strength. Increasing the thickness of the stiffener plate from 8 mm to 12 mm effectively delayed fatigue crack initiation and slowed down its propagation. Compared with fillet welds, full penetration welds extended the fatigue crack propagation life, but no significant improvement was implied for the initiation life.

### 3.4. Fatigue Crack Propagation Characteristics

Figure 10 shows beach marks on the fracture surfaces. *N* denotes the number of cycles of fatigue loading which excludes $\sum N_{\mathrm{BM},\;i}$, and $N_{I}$ is for the first dark band of the beach marks. Except for W3-2, radial steps were clearly visible on the fracture surface, which were overlaps of the cracks growing in slightly different planes. Therefore, it could be inferred that there were multiple crack nuclei. As for W3-2, the first dark band of the beach marks presented two adjacent semi-ellipses, suggesting two main crack nuclei. At the early stage of cracking, the shapes of beach marks were asymmetric, and their centers were located near the edge of the weld. This was probably due to the differences in propagation rates of cracking towards both sides of the stiffener plate. With the crack propagating, the shapes of the beach marks grew to be a single semi-ellipse. Their centers also gradually approached the centerline of the stiffener plate.

**Figure 11:**
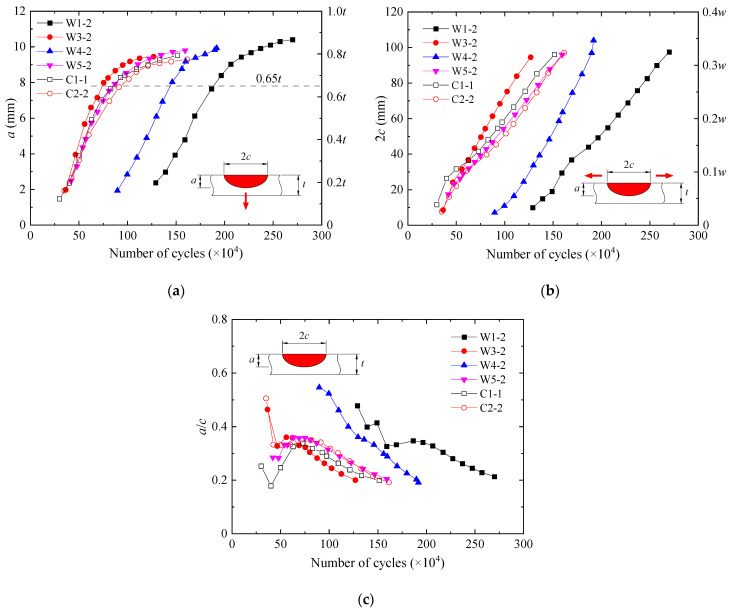
Fatigue crack propagation characteristics. (**a**) Crack depth. (**b**) Crack width. (**c**) Aspect ratio of crack.

Figure 11 shows the fatigue crack propagation characteristics of the specimens. The depth and width of cracks are denoted as *a* and 2*c*. The thickness and width of the deck plate are denoted as *t* and *w*. The cracking of W1-2 and W4-2 was much later than that of the other specimens. Nevertheless, the trends of crack propagating along the depth and width were coincident for all the specimens. The crack depth first increased nearly linearly, and after it reached about 0.65*t*, its growth rate slowed down. While the crack width kept increasing approximately linearly throughout. The aspect ratio *a*/*c* of W4-2 decreased monotonically. As for the other specimens, *a*/*c* fluctuated at the early stage of crack propagation, which was possibly related to multiple crack nuclei. Afterwards it went down, which indicated that the crack grew faster in the direction of the width than along the depth and that the shape of the crack tended to be slender. When the fatigue tests were finished, *a*, 2*c*, and *a*/*c* of the WS specimens reached (0.79~0.87)*t*, (0.32~0.35)*w*, and 0.19~0.21, respectively, and those of the CS specimens were (0.77~0.79)*t*, 0.32*w*, and 0.19~0.20. It should be noted that *a* of the first band of the beach marks was 1.5~2.5 mm and not accurate enough to track the very early crack initiation, for which further investigations were still needed. Moreover, the question of how steel grades and yield strengths influence the fatigue strength remains to be answered through microstructural and fractographic analysis [25].

## 4. Fatigue Strength Evaluation Based on *S*-*N* Curves

### 4.1. Nominal Stress and Hot Spot Stress Approaches

Nominal stress and hot spot stress are the reference stresses for fatigue assessment based on *S*-*N* curves. The nominal stress includes the stress concentration caused by macro-geometric discontinuity but excludes the contribution of welded joints to the stress increase. While the hot spot stress further considers the stress raising effect of welded joints, it disregards the nonlinear peak stress due to weld profiles. When the assessed structural details do not match any of the detail categories classified by the nominal stress approach, the hot spot stress approach may be used. For the specimens with the stiffener plate thickness of 12 mm, fatigue assessment was carried out using both the approaches.

**Figure 12:**
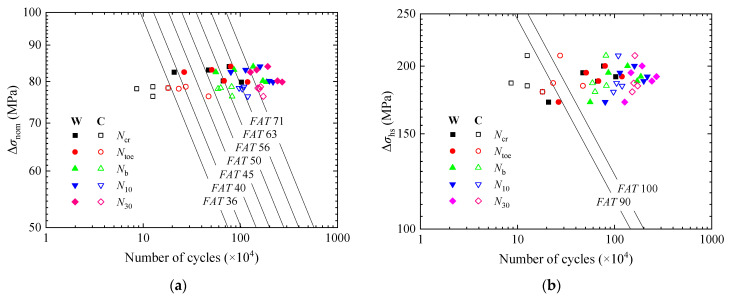
Comparison of fatigue data with *S*-*N* curves. (**a**) Nominal stress approach. (**b**) Hot spot stress approach.

The fatigue test results were compared with the *S*-*N* curves suggested by IIW, see Figure 12. $N_{\mathrm{cr}}$, $N_{\mathrm{toe}}$, $N_{b}$, $N_{10}$, and $N_{30}$ corresponded to the different states of the fatigue crack growing process from initiation to almost penetrating through the deck plate. As fatigue strength is directly related to the definition of fatigue failure, it should be settled which state is reasonable to define fatigue failure. Fatigue assessment was first conducted with the nominal stress approach, see Figure 12a. When $N_{\mathrm{cr}}$ and $N_{\mathrm{toe}}$ were used to define fatigue failure, the fatigue strength of the WS specimens was *FAT* 36 and *FAT* 40 respectively, but that of the CS specimens was lower than *FAT* 36. When $N_{b}$, $N_{10}$, and $N_{30}$ were used, the fatigue strength of both groups of the specimens reached *FAT* 50, *FAT* 56, and *FAT* 71 respectively. It is also found that the fatigue strength of the WS specimens is above that of the SM490 specimens [24]. However, the influence of the measurement position for the nominal stress still needs to be considered.

Then, the hot spot stress approach was adopted; see Figure 12b. When $N_{\mathrm{cr}}$ and $N_{\mathrm{toe}}$ were used as fatigue failure, the fatigue strength of both groups of the specimens was below *FAT* 90. When $N_{b}$ was used, it reached *FAT* 100. To make the fatigue failure definition in the approaches consistent with each other and provide the most conservative estimation, $N_{b}$ was taken to determine the fatigue strength, corresponding to the state where the crack reached the edges of the weld. Meanwhile, the width of the cracks was 31.5~44.1 mm, and the depth was 5.7~7.6 mm and less than 0.65 *t*, which meant the crack propagation rates along the depth would subsequently slow down and that it still took time for the crack to penetrate through the deck plate.

Therefore, the WS and CS specimens were classified as having the same fatigue strengths, which were *FAT* 50 and *FAT* 100, respectively, based on the nominal stress approach and the hot spot stress approach.

Statistical evaluation of the fatigue test data was further conducted to consider a safety margin. The *S*-*N* curve can be expressed as Equation (3):

(3) lgN=lgC−m⋅lgΔσ

where *N*, Δσ, and *m* denote the number of cycles, the stress range, and the slope of the curve, respectively; *C* is the constant reflecting fatigue resistance. Since the sample was small and the number of cycles in the fatigue tests was less than 10 million, *m* was taken as the value 3. A log-normal distribution was assumed. ${\left(\mathrm{lg}C\right)}_{i}$ was calculated from ${\left(N_{b},\Delta \sigma_{\mathrm{nom}}\right)}_{i}$ or ${\left(N_{b},\Delta \sigma_{\mathrm{hs}}\right)}_{i}$, where i is a rank number. With *x* denoting lgC, the characteristic value $x_{k}$ was obtained by Equation (4) [26], which is at 95% survival probability and calculated from the mean based on a two-sided confidence level of 75%. In Equation (4), $x_{m}$ and *Stdv* are the mean and the standard deviation of fatigue data respectively; *k* is a factor related to the sample size, survival probability, and confidence level of the mean. Then, fatigue strength was estimated using the *S*-*N* curve determined by $x_{k}$.

(4) $$x_{k}=x_{m}-k\cdot Stdv$$

Table 5 shows the results of the statistical evaluation. With the nominal stress approach and the hot spot stress approach, the fatigue strength of the WS specimens was 39 MPa and 74 MPa respectively, and that of the CS specimens was 44 MPa and 85 MPa. It was noticed that the WS specimens had greater $x_{m}$ but smaller $x_{k}$ than the CS ones did. This could be attributed to a larger scatter in the fatigue data of the WS specimens, and thus a more conservative estimation was made to satisfy the requirements of the survival probability and the confidence level of the mean.

### 4.2. Effective Notch Stress Approach

#### 4.2.1. Finite Element Method Modeling

**Figure 13:**
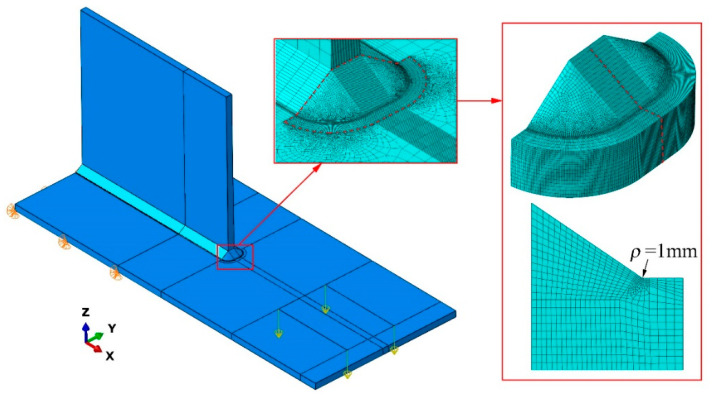
Finite element model for effective notch stress analysis.

The effective notch stress takes into account the stress raising effect of weld profiles by assuming a rounded shape with a reference radius at the weld toe or root. It cannot be measured directly. To obtain the effective notch stress, finite element models of the specimens with the stiffener plate thickness of 12 mm were established using ABAQUS [27], as shown in Figure 13.

**Figure 14:**
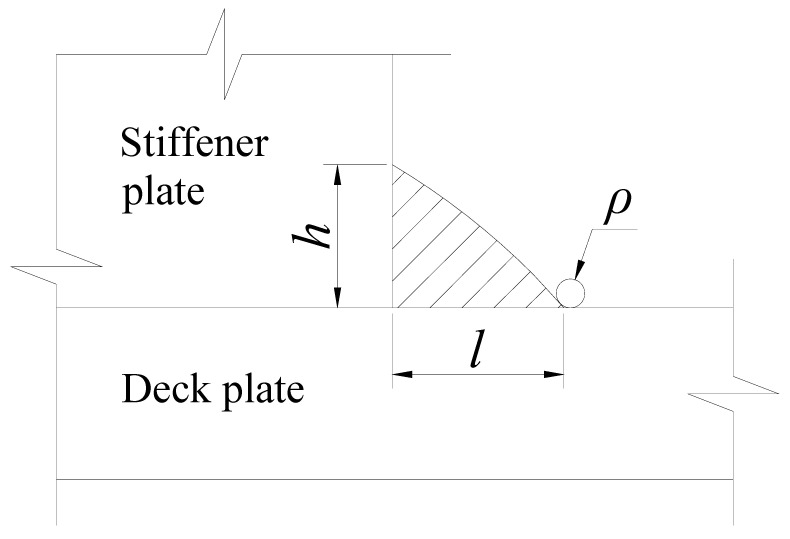
Geometric parameters of welds.

The geometric parameters of welds for effective notch stress analysis are illustrated in Figure 14, which includes the weld height *h*, the weld length *l*, and the reference radius *ρ* of the notch at the weld toe. The values of *h* and *l* are listed in Table 6, and *ρ* was equal to 1 mm. The Young’s modulus and the Poisson’s ratio were taken as 210 GPa and 0.3, respectively. Translational degrees of freedom were all restrained on the contact surface of the specimens with the pedestal. The applied loads were taken as ±1.58 kN to ensure the calculated nominal stress range reached 80 MPa. The part around the notch at the weld toe was finely meshed with the quadratic element C3D20R, where the element length was 0.1 mm of the notch arc and 0.2 mm of the radius and the adjacent tangent. This element size was corresponding to the mesh refinement recommendations for notch stress analysis by IIW [21]. The other parts were meshed with the linear element C3D8R, and the global element size was 5 mm. Besides, a mesh transition was used in the vicinity of the notch.

#### 4.2.2. Results and Analysis

**Figure 15:**
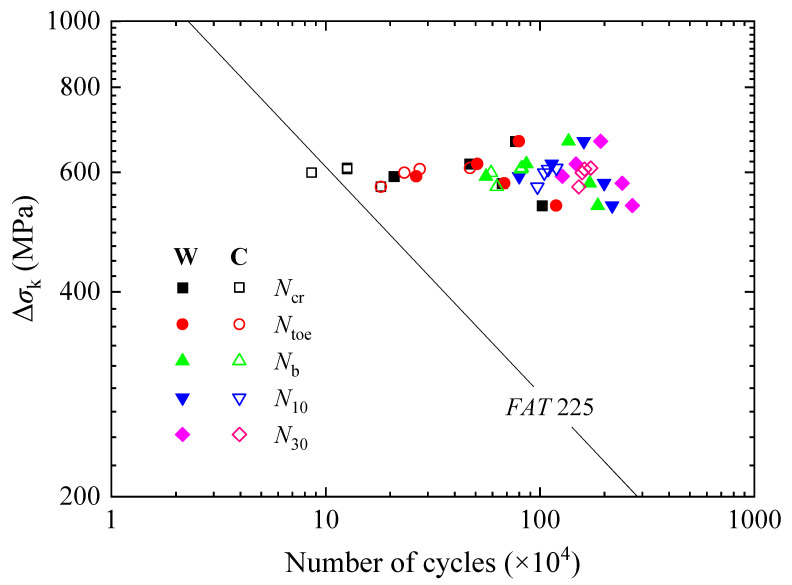
Comparison of fatigue data with *S*-*N* curve for effective notch stress range.

The results of effective notch stress analysis were compared with the *S*-*N* curve for a 1 mm reference radius by IIW, as shown in Figure 15. When $N_{\mathrm{cr}}$ was used to define fatigue failure, the fatigue strength of the WS specimens reached *FAT* 225, but that of the CS specimens was below *FAT* 225. When $N_{\mathrm{toe}}$ was used, the fatigue strength of both groups of the specimens reached *FAT* 225. To keep it consistent with the nominal stress approach and the hot spot stress approach, $N_{b}$ was taken as fatigue failure. The fatigue strength of *FAT* 225 was found to be still applicable to the WS and CS specimens.

Similar statistical evaluation was conducted as that in the nominal stress and hot spot stress analysis. The results are summarized in Table 7. The fatigue strength of the WS specimens and the CS specimens was 278 MPa and 304 MPa, respectively. Moreover, the differences in $x_{m}$ and $x_{k}$ between the two groups indicated a similar effect of the scatter on fatigue strength estimation as above mentioned.

## 5. Fatigue Crack Propagation Analysis by LEFM

### 5.1. Extended Finite Element Method Modeling

**Figure 16:**
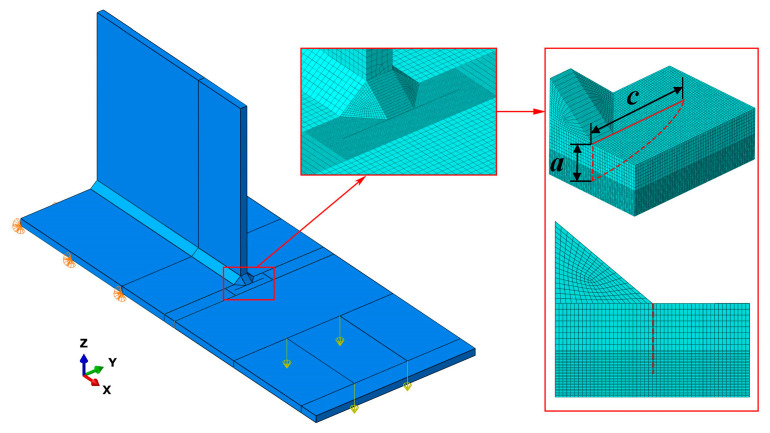
XFEM model.

Extended finite element method (XFEM) is an efficient technique for modelling cracks. In this method, enrichment functions incorporating both discontinuous fields and asymptotic fields near cracks are added to the finite element approximation, so that cracks are independent from the mesh and the complex meshing can be avoided [28]. As shown in Figure 16, XFEM models of the specimens with beach mark testing were established using ABAQUS to calculate the stress intensity factors *K* of the fatigue crack propagation process. Their material properties, boundary conditions, and loads were the same as those of the finite element models for effective notch stress analysis, but the notch at the weld toe was not rounded. The fatigue cracks were embedded into XFEM models, which were approximated as a series of semi-ellipses with a size of *a* and *c*. The linear element C3D8R was used to mesh all the parts. The crack domain was enriched, where the element size ranged from 0.1 to 0.3 mm in accordance with the crack size to ensure there were more than 10 contours. The global element size was 2.5 mm.

### 5.2. Fatigue Crack Propagation Rates

**Figure 17:**
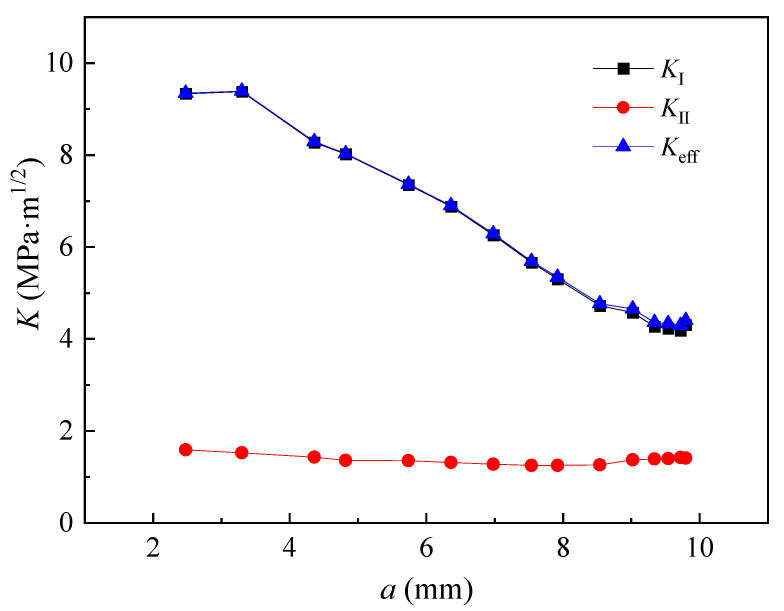
Stress intensity factors of W5-2 under load of 1.58 kN.

Fatigue cracks penetrating the deck plate were subject to both bending and shear stresses, which was a mixed mode. Their stress intensity factors $K_{\mathrm{eff}}$ were calculated by Equation (5) [29]:

(5) $$K_{\mathrm{eff}}=\sqrt[{4}]{K_{I}^{4}+8K_{\mathrm{II}}^{4}}$$

where $K_{I}$, $K_{\mathrm{II}}$, and $K_{\mathrm{eff}}$ denote the stress intensity factors for mode I, mode II, and mixed mode I and II, respectively. $K_{I}$ and $K_{\mathrm{II}}$ were obtained by XFEM analysis. W5-2 had the largest number of beach marks as well as the most specific information about fatigue cracking among the specimens. Its stress intensity factors under the load of 1.58 kN are illustrated in Figure 17. It was found that $K_{\mathrm{II}}$ was negligible in terms of the contribution to $K_{\mathrm{eff}}$. Therefore, the fatigue cracks were approximated as mode I cracks and $K_{\mathrm{eff}}$ were represented by $K_{I}$.

**Figure 18:**
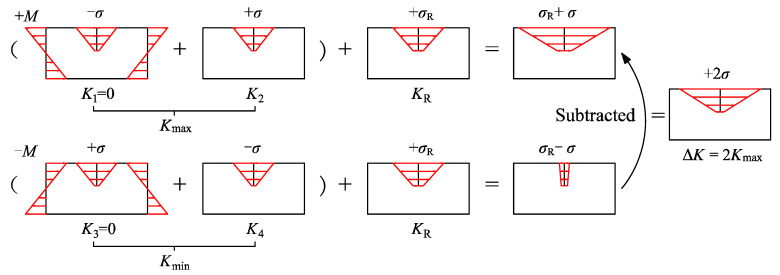
Calculating ΔK in tensile residual stress fields by superposition.

It is well-known that tensile residual stresses in welded joints can decrease crack closure and thus influence crack propagation. According to superposition principles, the stress intensity factor ranges ΔK considering tensile residual stresses were obtained by Equation (6) [30]:

(6) $$\Delta K=(K_{\max}+K_{R})-(K_{\min}+K_{R})$$

where $K_{\max}$ and $K_{\min}$ are the maximum and minimum stress intensity factors due to the external loads; $K_{R}$ is the stress intensity factor due to the residual stresses. For the fatigue cracks at the weld toe of vertical web stiffener to deck plate welded joints, $K_{\max}$ and $K_{\min}$ were split and ΔK was calculated as illustrated in Figure 18. The welding-induced tensile residual stresses cause the crack to remain open during fatigue loading [31]. Thus, $K_{\min}$ + $K_{R}$ > 0 and $K_{R}$ could be eliminated. ΔK was taken as 2$K_{\max}$.

**Figure 19:**
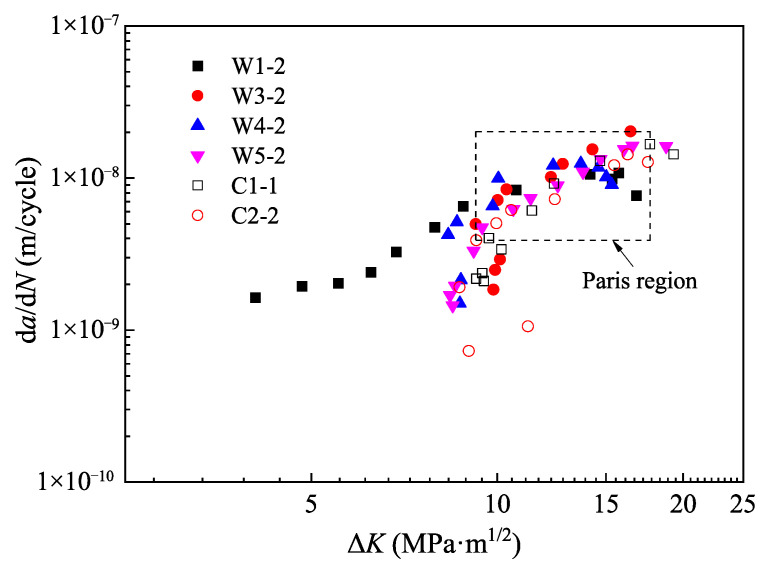
Fatigue crack propagation rates.

Seventh order polynomials were used to fit the crack propagation data ${\left(N,a\right)}_{i}$, and their derivatives were calculated to obtain the crack propagation rates d*a*/d*N*. Figure 19 shows the relationship between d*a*/d*N* and ΔK. As the d*a*/d*N* corresponding to the first and last dark bands of beach marks only considered one-sided data, they were not included in this figure.

### 5.3. Material Constants for LEFM

There is a period of stable crack growth during the fatigue crack propagation process, which is called Paris region. In this region, d*a*/d*N* and ΔK obey the Paris law and it can be expressed by Equation (7). *m* and *C* are constants related to the materials.

(7) lg(da/dN)=lgC+m⋅lg(ΔK)

Equation (7) indicates a linear relationship between d*a*/d*N* and ΔK in double logarithmic coordinates. Based on the principle that there were at least five consecutive data points for curve fitting and the coefficient of determination $R^{2}$ was greater than 0.95, a search was carried out from the first data point corresponding to the second dark band of beach marks to find the data points of Paris region. Then, the Paris region was marked with a dashed rectangle in Figure 19.

**Figure 20:**
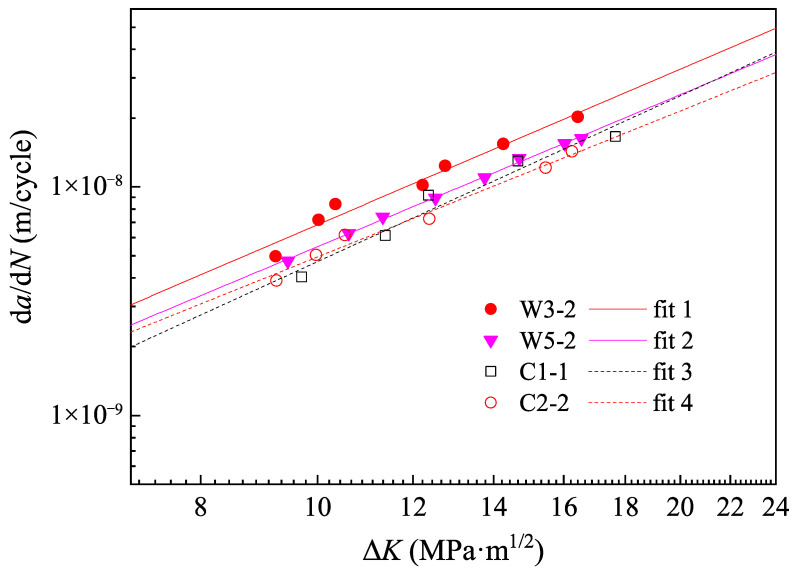
Fitted curves of Paris region.

Figure 20 shows the fitted curves for the data points in Paris region. W1-2 and W4-2 were excluded, since the scatter in their fatigue data was large and well fitted curves couldn’t be obtained. A similar situation was also reflected in Figure 11 that W1-2 and W4-2 were different from the other specimens in fatigue crack propagation characteristics. Table 8 shows the details of the fitted curves, including *m*, *C*, $R^{2}$, and the crack depth ranges of the data points in the Paris region. It was found that the material constants of the WS specimens were relatively close to those of the CS specimens. The mean values of *m* and *C* for the WS specimens were 2.24 and 3.56 × 10^−11^ respectively, and those for the CS specimens were 2.27 and 2.62 × 10^−11^. It should be noted that the mean value of *C* was calculated from lg*C*. Besides, the Paris region of the two groups was generally consistent, where the average crack depth range for the WS specimens was (0.35~0.72)*a*/*t* and that for the CS specimens was (0.31~0.70)*a*/*t*.

The material constants obtained from the fatigue data were compared with those of the standards and specifications; see Table 9. The difference of *C* was calculated by lg*C*. The values of *m* for the specimens were slightly smaller, which should be attributed to conservative requirements of the standards and specifications. Meanwhile, the values of *C* were relatively close and their difference was less than 3.3%. The standards and specifications were still applicable to the fatigue crack propagation evaluation of WS specimens.

## 6. Conclusions

To investigate the fatigue performance of vertical web stiffener to deck plate welded joints in weathering steel box girders, ten specimens of weathering steel (WS) and four specimens of plain carbon steel (CS) for comparison were tested by a vibratory fatigue testing machine and relevant numerical analysis was carried out. Conclusions can be drawn as follows:

1. The fatigue tests of vertical web stiffener to deck plate welded joints showed that the fatigue crack was initiated from the end weld toe of the deck plate accompanied by multiple crack nuclei, and subsequently propagated both along the thickness of the deck plate and in the direction perpendicular to the stiffener plate. Finally, it almost penetrated through the deck plate.
2. The fatigue crack initiation and propagation life of WS Q345qNH specimens was longer than that of CS Q345q specimens. The fatigue crack propagation life of WS Q345qNH specimens was longer than that of WS Q420qNH specimens, but the initiation life bore little relationship to the yield strength.
3. Increasing the thickness of the stiffener plate effectively delayed fatigue crack initiation and slowed down its propagation. Compared with fillet welds, full penetration welds extended the fatigue crack propagation life, but no significant improvement was implied for the initiation life.
4. The state where the crack reached the edges of the weld was taken as fatigue failure. The WS and CS specimens could be classified as having the same fatigue strengths by the nominal stress, hot spot stress, and effective notch stress approaches, which were *FAT* 50, *FAT* 100, and *FAT* 225, respectively. Meanwhile, their material constants for LEFM were relatively close to each other. The values of the material constant *m* for the specimens were slightly smaller than those of the recommendations by IIW, BS 7910, and JSSC, but the values of the material constant *C* were nearly the same. However, the beach marks are not accurate enough to track the early crack initiation in welded joints of WS and CS. New methods still need to be investigated to measure fatigue cracks during the early crack initiation.

## Figures and Tables

**Table 1:** Parameters of test specimens.

Specimen No.	Steel Grade [23]	Deck Plate Thickness t (mm)	Stiffener Plate Thickness b (mm)	Weld Type	Nominal Stress Range (MPa)	Stress Ratio
W1-1	Q345qNH	12	12	fillet weld	80	−1
W1-2						
W2-1	Q345qNH	12	12	full penetration weld	80	−1
W2-2						
W3-1	Q420qNH	12	12	fillet weld	80	−1
W3-2						
W4-1	Q420qNH	12	12	full penetration weld	80	−1
W4-2						
W5-1	Q345qNH	12	8	full penetration weld	80	−1
W5-2						
C1-1	Q345q	12	12	fillet weld	80	−1
C1-2						
C2-1	Q345q	12	12	full penetration weld	80	−1
C2-2						

**Table 2:** Chemical compositions of steel grades. (Mass fraction wt. %).

Steel Grade	C	Si	Mn	P	S	Als	Ni	Cu	Mo	Ti	Nb	Cr	Fe
Q345qNH	0.055	0.26	1.39	0.012	0.0035	0.034	Total 1.001						Bal.
Q420qNH	0.055	0.35	1.55	0.020	0.003	0.025	Total 1.187						Bal.
Q345q	0.150	0.30	1.46	0.013	0.0036	0.041	Total 0.182						Bal.

**Table 3:** Summary of fatigue test results.

Specimen No.	Δ*σ*_nom_ (MPa)	Δ*σ*_hs_ (MPa)	*K*_s_	*N*_toe_ (×10^4^)	*N*_b_ (×10^4^)	*N*_10_ (×10^4^)	*N*_30_ (×10^4^)	*N*_cr_ (×10^4^)	*N*_CP_ (×10^4^)	*R*_CP_ (%)
W1-1	79.7	199.7	2.51	run out	--	--	--	--	--	--
W1-2	79.9	191.4	2.40	119.0	186.4	217.1	270.0	102.3	167.7	62.1
W2-1	80.2	187.8	2.34	68.1	170.9	199.2	241.5	66.6	174.9	72.4
W2-2	79.8	196.8	2.47	run out	--	--	--	--	--	--
W3-1	83.1	194.6	2.34	51.0	86.5	113.6	147.1	46.9	100.2	68.1
W3-2	82.5	171.7	2.08	26.5	56.0	80.0	127.0	20.8	106.1	83.6
W4-1	81.2	187.5	2.31	run out	--	--	--	--	--	--
W4-2	84.0	200.2	2.38	79.7	136.1	159.9	191.9	76.7	115.2	60.0
W5-1	75.7	207.5	2.74	10.9	30.9	56.9	87.8	6.3	81.6	92.9
W5-2	79.6	180.5	2.27	34.5	57.1	98.7	159.4	15.1	144.3	90.5
C1-1	78.4	179.4	2.29	18.1	62.9	97.3	151.6	18.1	133.5	88.1
C1-2	78.2	186.2	2.38	23.3	59.1	104.7	157.1	8.6	148.5	94.5
C2-1	76.3	184.0	2.41	47.2	82.3	119.2	172.6	12.6	159.9	92.7
C2-2	78.7	209.3	2.66	27.5	81.3	109.1	161.5	12.6	149.0	92.2

**Table 4:** Comparison of fatigue life.

Structural Parameter	*N*_cr_	*N*_CP_	*N*_30_
Steel grade	W1 > C1	W1 > C1	W1 > C1
	W2 > C2	W2 > C2	W2 > C2
Yield strength	W1 > W3	W1 > W3	W1 > W3
	W2 < W4	W2 > W4	W2 > W4
Stiffener plate thickness	W2 > W5	W2 > W5	W2 > W5
Weld type	W1 > W2	W1 < W2	W1 > W2
	W3 < W4	W3 < W4	W3 < W4
	C1~C2	C1 < C2	C1 < C2

**Table 5:** Statistical estimation of fatigue strength.

Group No.	*k*	Nominal Stress Approach				Hot Spot Stress Approach			
		*x*_m_	*Stdv*	*x*_k_	*FAT*	*x*_m_	*Stdv*	*x*_k_	*FAT*
W	3.58	11.80	0.20	11.07	39	12.89	0.27	11.91	74
C	4.14	11.52	0.07	11.24	44	12.68	0.14	12.09	85

**Table 6:** Weld measurements.

Specimen No.	*h* (mm)	*l* (mm)	Specimen No.	*h* (mm)	*l* (mm)
W1-2	9.8	14.5	C1-1	7.8	11.3
W2-1	10.0	12.8	C1-2	10.2	12.2
W3-1	11.0	12.0	C2-1	11.2	12.6
W3-2	10.0	12.2	C2-2	11.5	12.8
W4-2	10.5	10.0			

**Table 7:** Statistical estimation of fatigue strength.

Group No.	*k*	Effective Notch Stress Approach			
		*x*_m_	*Stdv*	*x*_k_	*FAT*
W	3.58	14.39	0.21	13.63	278
C	4.14	14.17	0.10	13.75	304

**Table 8:** Summary of fitted curves (d*a*/d*N*: m/cycle; ΔK: $\mathrm{MPa}\cdot m^{1/2}$).

Specimen No.	Fitted Curve	*a* (mm)	*a*/*t*	*m*	*C*	*R* ^2^
W3-2	fit 1	3.95~8.66	0.33~0.72	2.26	3.75 × 10^−11^	0.9675
W5-2	fit 2	4.36~8.54	0.36~0.71	2.21	3.38 × 10^−11^	0.9978
C1-1	fit 3	3.89~8.29	0.32~0.69	2.41	1.83 × 10^−11^	0.9584
C2-2	fit 4	3.64~8.59	0.30~0.72	2.12	3.74 × 10^−11^	0.9806

**Table 9:** Comparison of material constants (d*a*/d*N*: m/cycle; ΔK: $\mathrm{MPa}\cdot m^{1/2}$).

Specimen No.	IIW [26]		Difference (%)		BS 7910 [32]		Difference (%)		JSSC [19]		Difference (%)	
	*m*	*C*	*m*	*C*	*m*	*C*	*m*	*C*	*m*	*C*	*m*	*C*
W3-2	3.0	1.65 × 10^−11^	−24.7	−3.3	2.88	2.7 × 10^−11^	−21.5	−1.3	2.75	2.7 × 10^−11^	−17.8	−1.3
W5-2			−26.3	−2.9			−23.3	−0.9			−19.6	−0.9
C1-1			−19.7	−0.4			−16.3	1.6			−12.4	1.6
C2-2			−29.3	−3.3			−26.4	−1.3			−22.9	−1.3

## Data Availability

Data are available upon request to the corresponding author.
